# Spatial Autocorrelations Between Potentially Suitable Habitats of *Aquatica leii* and Landscape Patterns Under Climate Change

**DOI:** 10.3390/insects17070717

**Published:** 2026-07-10

**Authors:** Chencheng Zheng, Dujuan Zhan, Yaqi Fang, Hao Li, Zhichao Huang, Xiaoli Fan

**Affiliations:** 1 College of Agriculture and Biotechnology, Lishui University, Lishui 323000, China; 240951320083@lsu.edu.cn (C.Z.); cynthiazhan@126.com (D.Z.); yaqifang@lsu.edu.cn (Y.F.); haoli@lsu.edu.cn (H.L.); 2 Modern Industrial College of Traditional Chinese Medicine and Health, Lishui University, Lishui 323000, China; 3 Zhejiang Liyong Ecology Tourism Development Company Limited, Lishui 323000, China; 504561072a@gmail.com

**Keywords:** *Aquatica leii*, climate change, MaxEnt model, potentially suitable habitat, landscape pattern, spatial autocorrelation

## Abstract

Climate change is reshaping species distributions worldwide, while landscape patterns can further influence habitat suitability and species persistence. Fireflies are important environmental indicators, given their high sensitivity to environmental disturbances. However, the effects of climate change and landscape structure on their future survival remain unclear. Here, we used the MaxEnt model to examine the current and predict the future potentially suitable habitats of *Aquatica leii*, an endemic aquatic firefly in China, under the SSP126, SSP245, and SSP585 climate scenarios. We also examined the spatial associations between potentially suitable habitats and landscape patterns using bivariate spatial autocorrelation analyses. The results showed that soil clay content, soil pH, annual mean temperature, slope, and distance to roads were the main factors influencing habitat suitability. Potentially suitable habitats were concentrated in western Zhejiang Province and are projected to expand under future climate scenarios. Potentially suitable habitats showed significant positive spatial associations with the number of patches, landscape shape index, Shannon’s diversity index, and Shannon’s evenness index. Associations with the largest patch index varied among scenarios, and were generally strengthened under future climate change. Overall, heterogeneous landscapes with diverse and complex habitat structures play an important role in supporting *A. leii* and should be considered in future conservation planning.

## Simple Summary

Climate change can influence the habitats of many animals and plants, and these habitats are also shaped by the surrounding landscape. Fireflies are key indicators of environmental quality because they are highly sensitive to environmental disturbances. However, how climate change and landscape structure affect their future survival remains unclear. *Aquatica leii* is an aquatic firefly found only in China, and its populations have declined rapidly in recent years. Here, we investigated how its suitable habitats may change under future climate conditions and the association of the habitats with different landscape patterns in the Zhejiang Province. Soil conditions and temperature are the main factors that determine habitat suitability. Suitable habitats are currently concentrated in western Zhejiang and are projected to expand under future climate scenarios. However, the most favorable habitats remain limited and become increasingly fragmented. Areas with diverse and complex landscape structures are more likely to support suitable habitats, with the relationships generally becoming stronger under climate change. Overall, these findings highlight the importance of maintaining heterogeneous landscapes with diverse habitat types for the conservation of *A. leii* and provide guidance for protecting other climate-sensitive aquatic insects.

## 1. Introduction

Climate change influences biological processes, phenology, and species distributions worldwide at an unprecedented rate [1]. Therefore, without effective mitigation and conservation measures, an increasing number of species are likely to face extinction. Insects constitute a major component of global biodiversity and play crucial roles in food webs and ecosystem functioning [2]. Shifts in their potential suitable habitats under climate change have received increasing attention. Prior studies have mainly focused on terrestrial insects, particularly agricultural and forestry pests with substantial economic impacts. Potentially suitable habitats for these pests, including *Batocera lineolata* [3], *Lymantria dispar* [4], and *Bactericera cockerelli* [5], have expanded under different climate change scenarios. In contrast, the responses of aquatic insects, which generally occupy relatively narrow ecological niches and strongly depend on aquatic environments, to climate change remain unclear. Most aquatic insects have biphasic life cycles, relying on aquatic habitats for larval development and adjacent terrestrial landscapes for adult survival and reproduction. They are highly sensitive to water temperature, water quality, and hydrological stability [6]. These ecological characteristics distinguish aquatic insects from terrestrial taxa and suggest that their responses to climate change cannot be inferred from studies on terrestrial insects. Climate-driven alterations in river runoff and aquatic system stability [7] may impose compounded ecological pressures, potentially resulting in rapid population declines [8]. Therefore, understanding how various aquatic insects respond to climate change is crucial to accurately assess the risk of biodiversity loss and develop targeted conservation strategies.

As climate change continues to reshape species distributions, understanding species distribution patterns has become fundamental to biodiversity conservation [9]. Species distribution models (SDMs), a key analytical tool, provide an effective approach for predicting potential distribution areas and identifying key environmental variables [10]. Various modeling approaches have been widely applied in species distribution modeling, including MaxEnt (maximum entropy model) [11], ecological niche factor analysis [12], and generalized linear models [13]. Among the available modeling approaches, MaxEnt, which is based on machine learning and the maximum entropy principle, is one of the most widely used methods. Compared with conventional species distribution modeling techniques, it has superior predictive accuracy and robustness, particularly when handling small sample sizes and complex nonlinear ecological relationships [14]. This model is widely used to predict potentially suitable habitats for insect species. Previous studies that have used MaxEnt have indicated a poleward shift in the potential distribution of *Monochamus alternatus* [15], successfully delineated the potentially suitable habitats of *Ectomyelois ceratoniae* [16], and identified expanding suitable habitats for *Closterocerus chamaeleon* [17]. However, most prior studies have focused on large-scale predictions driven by climatic variables rather than on habitats that species can occupy and persist in [18]. Climatic variables alone are often insufficient to fully explain the spatial distribution patterns of aquatic insects that depend on aquatic environmental conditions. Landscape structure at the regional scale can regulate species distributions by affecting water system integrity, habitat quality, and dispersal processes [19]. Accordingly, some researchers have recommended incorporating landscape metrics into species distribution models to better capture the constraints imposed by habitat fragmentation under climate change [20,21]. For aquatic insects characterized by biphasic life cycles and dual dependence on aquatic and terrestrial habitats, landscape structure has a stronger constraint on habitat suitability than on strictly terrestrial taxa, as it shapes stream habitat integrity and the connectivity between larval and adult resources. Nevertheless, these approaches have rarely been applied to the endangered endemic aquatic firefly *Aquatica leii*.

Aquatic insects often occupy narrow ecological niches and have complex biphasic life cycles. Therefore, they are particularly vulnerable to global environmental change. Among them, fireflies strongly depend on both aquatic and terrestrial habitats and are widely regarded as important environmental indicators [22]. This makes them an ideal model group for investigating the interactive effects of climate and landscape changes. The endemic Chinese species *Aquatica leii* belongs to the class Insecta, order Coleoptera, family Lampyridae, and genus *Aquatica*. Its larvae inhabit shallow streams with clear water and slow flow and are highly sensitive to water conditions changes [23]. After emergence, adults occupy terrestrial habitats surrounding streams, reflecting a dual dependence on aquatic and terrestrial environments [24]. Their complex life cycle suggests that the species may face more intricate ecological risks of climate change than do insects restricted to a single habitat. Continuous population declines have been reported, with some local populations approaching extinction [25]. These rapid declines reflect the urgent need to predict potentially suitable habitats and assess habitat conditions, which are essential for developing effective conservation strategies. 

Situated in southeastern China, the Zhejiang Province is characterized by a complex topography, extensive mountainous and hilly landscapes, dense river networks, and a humid climate. This makes it a crucial distribution area for *A. leii*. Rapid urbanization. However, land-use change have intensified landscape fragmentation in the region [26], and climate change has increased the temporal and spatial variability of precipitation [27]. These combined pressures highlight the need to examine dynamic changes in the potentially suitable habitats of *A. leii* and their coupling with landscape patterns. A national scale MaxEnt based prediction of the potential distribution of *A. leii* in China has been conducted [28], focusing primarily on climatic suitability. However, this framework has limited capacity to represent fine scale spatial heterogeneity and landscape level processes that may influence habitat suitability at regional scales. The relationships between habitat suitability and landscape structure remain insufficiently understood when examined at finer spatial resolutions. Thus, further research is needed to improve the spatial understanding of habitat environment relationships in regional contexts. However, such studies remain limited, representing a crucial gap that constrains regional biodiversity conservation planning in the region.

Therefore, we sought to accomplish three objectives in this study: (1) identify the key environmental variables that shape the spatial distribution of potentially suitable habitats of adult *A. leii*; (2) project the dynamics of its potentially suitable habitats under current and future climate scenarios; and (3) analyze the spatial associations between potentially suitable habitats and landscape patterns using the bivariate Global Moran’s I and Local Moran’s I. The findings provide a basis for evaluating the effects of climate change on the distribution and persistence of *A. leii*. They support the development of conservation and management strategies, identifying priority areas and contributing to a deeper understanding of the adaptive responses of climate-sensitive aquatic insects to global change, guiding targeted conservation actions.

## 2. Materials and Methods

### 2.1. Reporting and Ethics Standards

In this study, we followed relevant reporting guidelines for species distribution modeling, specifically the Overview-Data-Model-Assessment-Prediction protocol [29]. We used non-invasive field survey methods (i.e., non-intrusive investigation without contact or capture of animals). No animal specimens were collected, handled, or harmed during the study. Field investigations were conducted with permission from local forestry authorities and land managers and complied with applicable local environmental regulations and biodiversity conservation requirements.

### 2.2. Study Area

**Figure 1:**
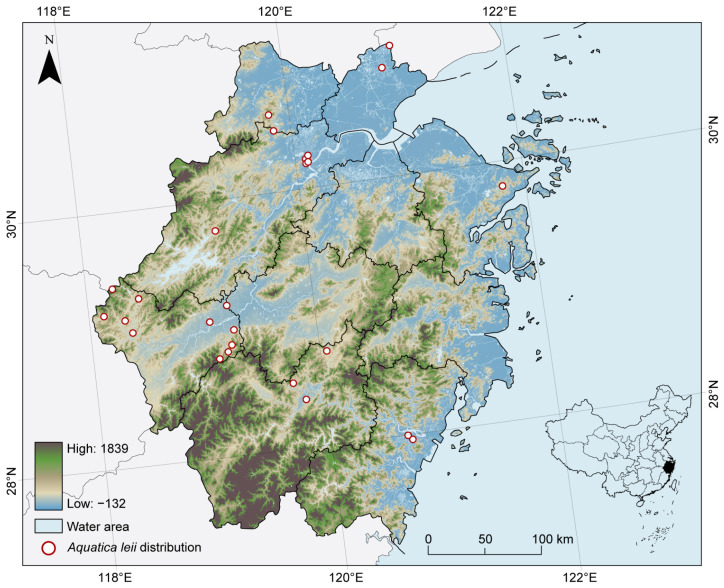
Location of the valid distribution points of *Aquatica leii* in the Zhejiang Province, China.

The Zhejiang Province lies on the southeastern coast of China, south of the Yangtze River Delta, between 118°01′–123°10′ E and 27°06′–31°11′ N and covers approximately 105,500 km^2^. The terrain slopes from west to east, with mountainous and hilly regions in the west characterized by dense stream networks. Meanwhile, the eastern coastal plains are highly urbanized (Figure 1). The province comprises mountainous areas (74.6%), plains (20.3%), and surface water (5.1%), resulting in a relatively high river network density. The region has a subtropical humid monsoon climate, with annual precipitation ranging from 1100 to 2000 mm and a mean annual temperature of 15–18 °C. The forest vegetation is dominated by mid-subtropical evergreen broad-leaved forests [30]. Notably, Zhejiang has experienced rapid urbanization, resulting in increasingly prominent ecological and environmental issues that challenge the sustainable development of landscape ecosystems.

### 2.3. Occurrence Records for A. leii

We compiled occurrence records of adult *Aquatica leii* in the Zhejiang Province based on our previous studies [28], and supplemented them with additional field survey data. After data integration and verification, a total of 30 unique occurrence records were obtained for analysis (Table 1). To mitigate spatial autocorrelation among samples and reduce the risk of model overfitting caused by clustered occurrence points, we performed a standardized screening procedure in ArcGIS Pro 3.1.6. We first removed duplicate records falling within the same raster cell of the resampled environmental layers to ensure each environmental unit contributed only one independent sample. We then excluded invalid points located within large water bodies or with coordinates inconsistent with the documented distribution range of the species. We applied a spatial rarefaction with a 1-km distance threshold to remove geographically adjacent clustered points, so that all retained sites had independent environmental information.

After screening, 26 valid distribution points were retained for the final MaxEnt modeling (Figure 1). These points span a longitudinal range of 118°08′–123°48′ E and a latitudinal range of 27°58′–31°01′ N, covering all known major distribution areas of *A. leii* in western Zhejiang, including Hangzhou, Quzhou, Lishui and Jinhua. Distributed along stream networks, these sites cover the full environmental gradient of the species’ core habitat with relatively low spatial aggregation. This sample size is sufficient for MaxEnt, which performs reliably with ≥10 occurrence records, especially after spatial filtering [14].

### 2.4. Data on Environmental Variables

Based on previous studies on the habitat characteristics of adult *A. leii*, we selected four environmental variable categories to model its potentially suitable habitats—bioclimatic, soil, topographic, and anthropogenic disturbance variables. We obtained topographic variables and the normalized difference vegetation index (NDVI) from the Resource and Environment Science Data Center of the Chinese Academy of Sciences. We derived climate data from the BCC-CSM2-MR model developed by the Beijing Climate Center under CMIP6 [31]. To represent future climate conditions in the 2050s and 2070s, we selected three climate scenarios under the shared socioeconomic pathway (SSP) framework: SSP126, SSP245, and SSP585. We obtained nighttime light data from the harmonized Defense Meteorological Satellite Program-Operational Linescan System and Suomi National Polar-Orbiting Partnership Visible Infrared Imaging Radiometer Suite datasets available on the Harvard Dataverse platform [32]. We obtained soil data from version 2.0 of the Harmonized World Soil Database raster product released by the Food and Agriculture Organization of the United Nations (https://gaez.fao.org/pages/hwsd, accessed on 2 May 2026). We obtained data on rivers, roads, and settlements from the 1:1,000,000 national fundamental geographic database of China for 2022 (http://www.ngcc.cn, accessed on 2 May 2026). Then, we calculated Euclidean distance layers for rivers, roads, and settlements using ArcGIS Pro 3.1.6, where distances were calculated from each raster cell center to the nearest river channel, road segment, or settlement boundary within the study area, respectively. Subsequently, we resampled and spatially aligned raster datasets to a common resolution, extent, and projection using ArcGIS, and then clipped them to the administrative boundary of Zhejiang Province under the World Geodetic System 1984 coordinate system to maintain spatial uniformity across all the environmental variables.

**Figure 2:**
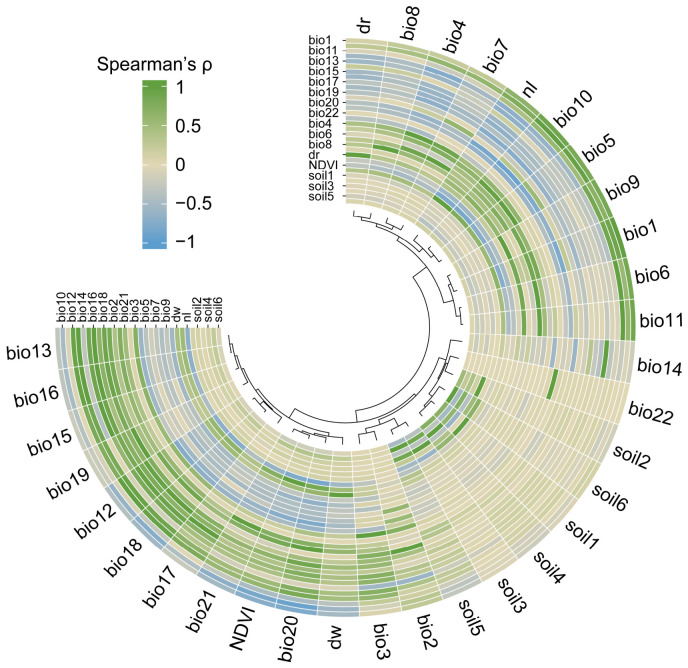
Correlation analysis of 32 environmental variables. Colors indicate the strength and direction of pairwise correlations among variables, with blue representing negative correlations and green representing positive correlations. Abbreviations are as follows: annual mean temperature (bio1), diurnal temperature range (bio2), isothermality (bio3), coefficient of seasonal variation in temperature (bio4), mean temperature of the wettest quarter (bio8), precipitation of the driest month (bio14), precipitation seasonality (bio15), precipitation of the driest quarter (bio17), precipitation of the coldest quarter (bio19), slope (bio21), aspect (bio22), normalized difference vegetation index (NDVI), nighttime light data (nl), distance to roads (dr), distance to water sources (dw), soil sand content (soil1), soil silt content (soil2), soil clay content (soil3), soil reference bulk density (soil4), and soil pH (soil6).

We conducted a variable selection procedure to reduce multicollinearity and redundancy among environmental variables [33]. We calculated Spearman’s correlation coefficient (ρ) among the 32 environmental variables to assess collinearity (Figure 2). When the absolute correlation coefficient exceeded 0.8, we removed one of the two variables to reduce redundancy. For each pair of highly correlated variables, we retained the variable with higher permutation importance derived from a single preliminary MaxEnt models [34]. Finally, 20 environmental variables were retained for the final MaxEnt model (Table 2).

### 2.5. MaxEnt Model Construction and Delineation of Potentially Suitable Habitats

We input the processed occurrence records as presence points and filtered environmental variables as predictors into the MaxEnt model. We ran the model using a random seed and randomly selected 25% of the occurrence records as test data. We replicated the model 10 times using the bootstrap method. We used the Jackknife test and response curves to assess the variable importance and model performance. We generated the model outputs in a logistic format and exported them as ASCII raster files. Next, we evaluated model performance using the area under the receiver operating characteristic curve (AUC), with values ranging from 0 to 1. AUC values of 0.7–0.9 indicate good performance, and values >0.9 represent excellent performance [35].

We conducted spatial analysis of the MaxEnt outputs using ArcGIS. We classified the predicted results into four categories using the Jenks natural breaks classification method: unsuitable (0–0.149), low-suitability (0.149–0.306), moderate-suitability (0.306–0.516), and high-suitability habitats (0.516–0.996). We calculated the area proportions of each suitability class based on the raster attribute tables. We used the SDMtoolbox Pro 0.9.1 to perform raster difference analysis and quantify the stable, expanded, and contracted areas of potentially suitable habitats. We then generated the spatiotemporal distribution maps of potentially suitable habitats.

### 2.6. Landscape Metrics Selection

We obtained land-use data used for landscape pattern analysis from Figshare, which provides simulations of land-use patterns under multiple climate scenarios from 2020 to 2100 [36]. These datasets are scenario-based projections consistent with the SSP framework and are aligned with CMIP6 climate scenarios used in this study, ensuring consistency between land-use and climate forcing data. We calculated landscape metrics using Fragstats 4.2, a widely used tool for quantifying landscape structure across various land-use types. Considering the limited applicability of patch-level metrics in this study, we selected five representative landscape-level indices: number of patches (NP), largest patch index (LPI), landscape shape index (LSI), Shannon’s diversity index (SHDI), and Shannon’s evenness index (SHEI) (Table 3). These indices capture key aspects of landscape composition and configuration and are sensitive to the alterations in insect habitats alterations [37]. We assessed pairwise Pearson correlations among the five selected landscape metrics to evaluate their internal collinearity (Table 4). Despite strong covariation across metrics, each index reflects a distinct structural dimension of landscape patterns and provides independent ecological information for subsequent bivariate spatial autocorrelation analysis.

### 2.7. Bivariate Spatial Autocorrelation Analysis

We applied a bivariate spatial autocorrelation approach to explore the spatial associations among the variables. Originally proposed as an extension of spatial autocorrelation analysis, this technique assesses the relationship between a given variable at one location and another in neighboring locations [38]. It has been widely employed in landscape ecology, environmental science, and geography [39]. Herein, we generated 4819 grid cells of 5 × 5 km as the basic analytical units. To quantify the spatial associations between potentially suitable habitats and landscape patterns across the study area, we extracted the mean habitat suitability values and corresponding landscape metrics for each grid cell using zonal statistics in ArcGIS. We calculated Global Moran’s I and Local Moran’s I, also known as Local Indicators of Spatial Association (LISA), using GeoDa. The equations used are as follows:

(1) $$\mathrm{Global}\;\mathrm{Moran}’s\;I\;=\;\frac{\sum_{i=1}^{n}\sum_{j=1}^{n}w_{\mathrm{ij}}(x_{i}\;-\bar{x})(y_{j}\;-\;\bar{y})}{S_{x}S_{y}\sum_{i=1}^{n}\sum_{j=1}^{n}w_{\mathrm{ij}}}$$

(2) $$\mathrm{Local}\;\mathrm{Moran}’s\;I=\frac{\left(x_{i}\;-\;\bar{x}\right)}{S_{x}}\sum_{j=1}^{n}w_{\mathrm{ij}}\frac{\left(y_{j}\;-\;\bar{y}\right)}{S_{y}}$$

where n is the number of spatial units; x_i_ is the average habitat suitability value of unit i; y_j_ is the landscape metric of unit j; w_ij_ is the spatial weight representing the adjacency relationship between units; and S_x_ and S_y_ are the standard deviations of the habitat suitability and landscape metrics, respectively. Global Moran’s I range from −1 to 1, with values > 0 indicating a positive spatial association between the potential suitable habitats and the corresponding landscape metric, values < 0 indicating a negative correlation, and values = 0 indicating no spatial association and a random distribution pattern. Local Moran’s I indicate the strength and direction of spatial associations between each unit and its neighboring units. For both current and future periods, based on the LISA framework implemented in GeoDa with a significance threshold of *p* < 0.05, local spatial associations were classified into five categories: high–high, low–low, high–low, low–high, and not significant.

## 3. Results

### 3.1. Key Environmental Variables

**Figure 3:**
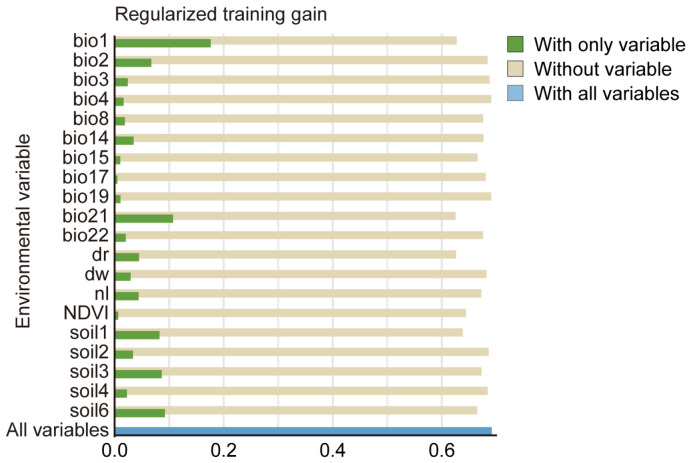
Importance of environmental variables from the jackknife analysis. Climatic variables include annual mean temperature (bio1), diurnal temperature range (bio2), isothermality (bio3), temperature seasonality (bio4), mean temperature of the wettest quarter (bio8), precipitation of the driest month (bio14), precipitation seasonality (bio15), precipitation of the driest quarter (bio17), and precipitation of the coldest quarter (bio19). Soil variables include soil clay content (soil3), sand content (soil1), silt content (soil2), reference bulk density (soil4), and soil pH (soil6). Topographic variables include slope (bio21) and aspect (bio22). Additional predictors include normalized difference vegetation index (NDVI), nighttime light data (nl), distance to roads (dr), and distance to water sources (dw).

**Figure 4:**
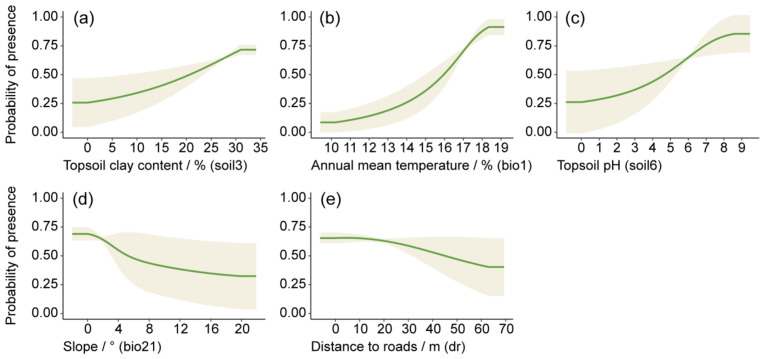
Response curve of the key environmental variables. Green lines indicate the mean probability of presence, and shaded areas represent the 95% confidence intervals. (**a**–**e**) Soil clay content (soil3), annual mean temperature (bio1), soil pH (soil6), slope (bio21), and distance to roads (dr), respectively.

The MaxEnt model achieved a mean test AUC of 0.919 ± 0.026, indicating good and stable predictive performance. However, the results should be interpreted with caution given the limited sample size. Based on the jackknife test (Figure 3) and response curves (Figure 4), soil clay content (soil3), annual mean temperature (bio1), soil pH (soil6), slope (bio21), and distance to roads (dr) were the most influential variables (Table 2). Their corresponding percentage contributions were 19.2%, 16.6%, 10.5%, 8.6%, and 7.4%, with permutation importance values of 15.0%, 9.8%, 2.4%, 6.5%, and 11.1%, respectively. These variables accounted for a cumulative contribution of 62.3%, indicating that they were the dominant drivers of habitat suitability for *A. leii*. Based on these dominant environmental variables, we further analyzed the spatial distribution of potentially suitable habitats under current and future climate scenarios.

### 3.2. Potential Suitable Habitats of A. leii Under Various Periods and Climate Scenarios

**Figure 5:**
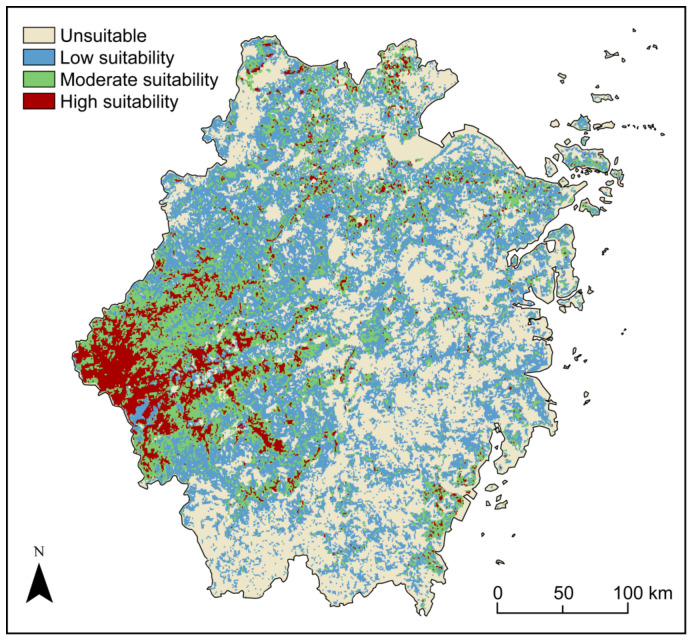
Current potentially suitable habitats of *Aquatica leii* in the Zhejiang Province.

To better understand the impacts of climate change on habitat dynamics, we analyzed the current distribution patterns of potentially suitable habitats and compared the patterns against future projections under different climate scenarios. Under current conditions, the potentially suitable habitats of *A. leii* are mainly distributed in western Zhejiang Province (Figure 5), covering approximately 58,600 km^2^. This accounts for 55.54% of the provincial land area. Within this area, highly suitable habitats cover approximately 6500 km^2^ (6.16%), moderately suitable habitats cover 17,200 km^2^ (16.30%), and low-suitable habitats cover 34,900 km^2^ (33.08%). Moderate- and low-suitability habitats are relatively fragmented. Meanwhile, large, contiguous, highly suitable habitats were mainly concentrated in Quzhou. Highly suitable habitats were mainly located in the Majinxi River Basin in Kaihua County, the Changshan Port Basin in Changshan County, the Yuelianghu Basin in Jiangshan, Xianxia Lake and the Qujiang River Basin in the Qujiang District of Quzhou, the Songyangxi River Basin in Songyang County, the Jiulong Wetland area in the Liandu District, the Longquanxi River Basin in Yunhe County of Lishui, the Qiandao Lake region in Chun’an County of Hangzhou, the Lanjiang River Basin in Lanxi, and the Shafan and Jinlan reservoirs in the Wucheng District of Jinhua. Low-suitability habitats exhibit a spatial pattern similar to that of moderately suitable habitats. In addition to extending outward from highly suitable habitats, they are distributed in the Qiantang River Basin in Hangzhou, Nanhu area in Jiaxing, Sanyang Wetland in Wenzhou, and Laolongxi River Basin in Huzhou.

**Figure 6:**
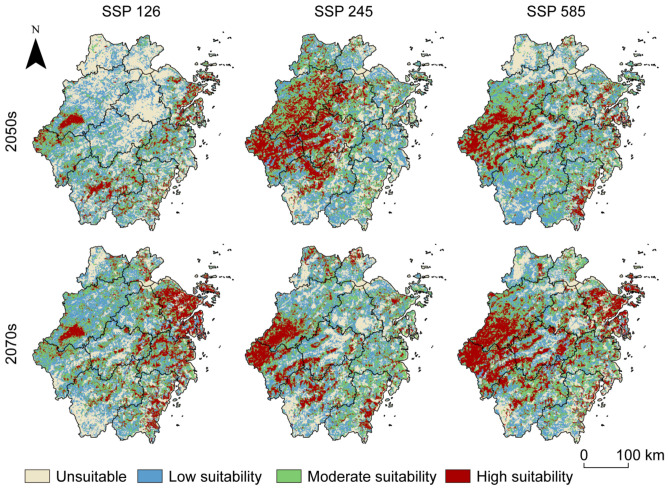
Potential suitable habitats of *Aquatica leii* under future climate scenarios (SSP126, SSP245, and SSP585).

Under the future climate scenarios, the total area of potentially suitable habitats for *A. leii* shows an increasing trend in the Zhejiang Province (Table 5; Figure 6). Under the SSP126 scenario, potentially suitable habitats expand in eastern Ningbo, eastern Taizhou, eastern Wenzhou, and the Qiandao Lake region in Hangzhou. In contrast, suitability decreases in Quzhou, leading to a net increase of approximately 15,100 km^2^. Under the SSP245 scenario, highly suitable habitats increase in northern Lishui, northwestern Quzhou, and the Qiandao Lake region, with a pattern of initial expansion followed by contraction in Jinhua. This results in an increase of approximately 12,700 km^2^. Under the SSP585 scenario, highly suitable habitats initially expand in Quzhou, Hangzhou, and Jinhua, and subsequently extend to Ningbo and Zhoushan, leading to a total increase of approximately 16,300 km^2^.

### 3.3. Global Moran’s I Between Potentially Suitable Habitats and Landscape Metrics

**Figure 7:**
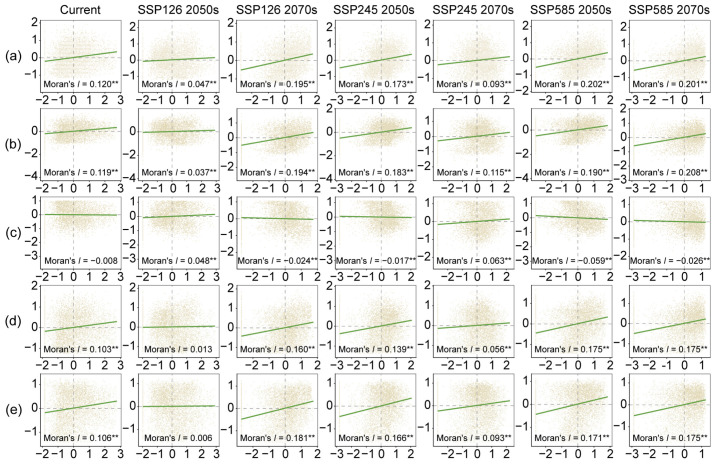
Moran scatter plots showing the bivariate spatial autocorrelation between the potential suitable habitats of *Aquatica leii* and five landscape pattern indices across the Zhejiang Province under different climate scenarios (SSP126, SSP245, and SSP585). (**a**–**e**) Number of patches (NP), landscape shape index (LSI), largest patch index (LPI), Shannon’s diversity index (SHDI), and Shannon’s evenness index (SHEI), respectively. In each plot, the x-axis represents the standardized habitat suitability value, and the y-axis represents the spatially lagged value of the corresponding landscape metric, calculated as the standardized mean value of neighboring units. ** *p* ≤ 0.01.

The Global Moran’s I results (Figure 7) indicate that most spatial associations between the potentially suitable habitats of *A. leii* and landscape metrics are statistically significant (*p* < 0.01). Exceptions are observed for LPI in the current period and SHDI and SHEI under the SSP126 scenario in the 2050s. Potentially suitable habitats exhibit considerable spatial associations with the landscape metrics. Negative correlations were observed between potentially suitable habitats and LPI under SSP245 and SSP585 in the 2050s and under SSP126 and SSP585 in the 2070s. In contrast, positive correlations were observed with all the other metrics. Across climate scenarios, the absolute values of the correlation coefficients increased, indicating progressively stronger spatial associations over time.

Moran scatterplots further support these patterns, indicating a generally stable spatial structure across periods, with points distributed across all four quadrants. For the LPI, most points are concentrated in the second and fourth quadrants. This corresponds to negative Moran’s I values and indicates a clear negative spatial association with potentially suitable habitats. In contrast, Moran’s I values for NP, LSI, SHDI, and SHEI are positive, with most points clustered in the first and third quadrants, indicating positive spatial associations.

### 3.4. Local Moran’s I Between the Potentially Suitable Habitats and Landscape Metrics

**Figure 8:**
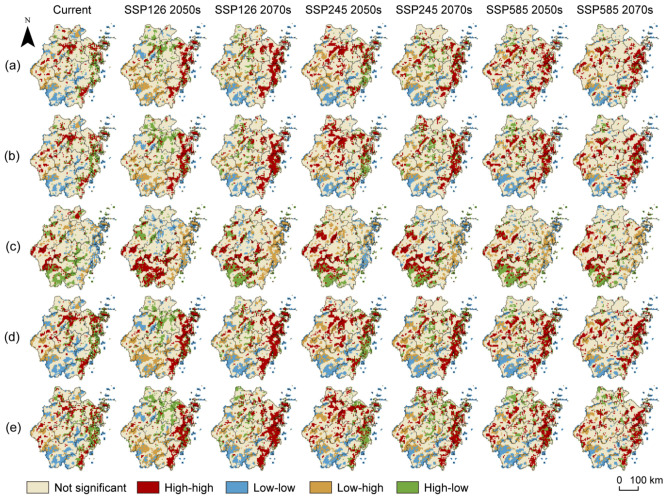
Local Indicators of Spatial Association (LISA) cluster maps depicting the local bivariate spatial autocorrelation between the potential suitable habitats of *Aquatica leii* and five landscape pattern indices across the Zhejiang Province under SSP126, SSP245, and SSP585 climate scenarios. (**a**–**e**) Number of patches (NPs), landscape shape index (LSI), largest patch index (LPI), Shannon’s diversity index (SHDI), and Shannon’s evenness index (SHEI), respectively.

We performed LISA for the 2050s and the 2070s to visualize the spatial clustering patterns (Figure 8). High–high clusters indicate areas where high habitat suitability is linked to high landscape metric values, whereas high–low clusters indicate areas where high habitat suitability is associated with low landscape metric values, similar to other cluster types. For LPI, during periods with negative correlations, high–low clusters are mainly distributed in Lishui and Hangzhou, whereas low–high clusters are concentrated in the eastern coastal regions, including Ningbo, Taizhou, and Wenzhou. During periods of positive correlations, high–high clusters are primarily located in Lishui and Hangzhou, whereas low–low clusters are concentrated in Zhoushan and Taizhou. The spatial patterns of the NP, LSI, SHDI, and SHEI are generally consistent. Under the current conditions, high–high clusters are primarily situated in the border area between Hangzhou and Shaoxing, whereas low–low clusters are primarily distributed in Lishui and western Hangzhou. Under the SSP126 and SSP585 scenarios, high–high clusters are primarily concentrated in the eastern coastal regions, including Ningbo, Taizhou, and Wenzhou, whereas low–low clusters are mainly distributed in Lishui. Under SSP245, high–high clusters are initially concentrated in Hangzhou and Jinhua and then shift to the eastern coastal regions, whereas low–low clusters primarily remain in Lishui.

Based on the LISA cluster statistics (Table 6), we compared changes in the number of clustered spatial units across periods and scenarios. The number of low-value clusters remained relatively stable, whereas high-value clusters exhibited noticeable fluctuations.

## 4. Discussion

The dynamic changes in the potentially suitable habitats of *A. leii* are jointly shaped by complex interactions between environmental variables and landscape patterns. Across periods and climate scenarios, the potentially suitable habitats of *A. leii* show varying responses to different landscape metrics. Meanwhile, the effects of landscape patterns on potentially suitable habitats generally increase with increasing climate change intensity. We integrated species distribution modeling and spatial autocorrelation analysis to identify the key environmental variables that affect habitat suitability, analyze the dynamic changes in potentially suitable habitats, and identify their spatial associations with landscape patterns. Based on key environmental variables and the spatial correlations between potentially suitable habitats and different landscape metrics, we further discuss implications for the conservation and management of *A. leii*.

### 4.1. Key Environmental Variables That Affect the Potentially Suitable Habitats of A. leii

The environmental variables identified by the model reflect the habitat requirements of *A. leii* across its full life cycle. As all occurrence records were derived from adult surveys, these variables primarily characterize terrestrial habitats for adult survival and reproduction, which are spatially associated with adjacent aquatic habitats supporting larval development. Multiple factors affect species distributions, among which soil variables play a crucial role in shaping insect distribution patterns under climate change [40]. Soil clay content (soil3) and soil pH (soil6) were the primary soil-related key environmental variables that influenced the potentially suitable habitats of *A. leii*, with a combined contribution of 29.7%. This result is consistent with those of previous studies, which have shown that soil conditions are crucial drivers of firefly distribution patterns [41]. Under climate change, soil factors may have a stronger influence on aquatic insects during their terrestrial larval stages than other climatic variables. Response curves indicated that a clay content of >30% was favorable for *A. leii* distribution. This may be attributed to improved water retention capacity and microclimatic stability of terrestrial habitats with higher clay content, which benefits adult activity and pupal development, and helps to maintain hydrological conditions of adjacent streams for larval growth. Soil pH > 7 was linked to higher habitat suitability. Alkaline soil conditions can provide suitable pupal substrates and support diverse invertebrate communities in riparian zones, which contributes to stable terrestrial habitats for adult survival [42]. These findings reflect the importance of soil conditions in maintaining terrestrial habitats that support the persistence of *A. leii* populations.

Annual mean temperature (bio1) contributes 16.6% to the model, with suitable conditions occurring at >17 °C. This indicates that temperature is a key limiting factor for firefly survival [43]. Lower temperatures may constrain population establishment by decreasing developmental rates or prolonging life cycles. In contrast, precipitation-related variables showed relatively low contributions, despite *A. leii* being an endemic species with fully aquatic larvae that are highly sensitive to water conditions [23]. This pattern may be attributed to the fact that all occurrence records in this study were collected from the adult stage. Precipitation primarily influences the aquatic larval stage by regulating the hydrological conditions of water bodies and has a weaker limiting effect on the terrestrial distribution of adults. To avoid underestimating the potential effects of relevant environmental variables, future studies should incorporate field survey data of the larval stage to analyze the environmental requirements of *A. leii* across its entire life cycle.

Topographic factors are among the key environmental variables that affect the potential distribution of fireflies. Suitable conditions are linked to slopes ranging from 0° to 4° and distances to roads of 0–20 m. Gentle slopes support water retention and sediment accumulation and are often linked to lower light pollution levels [44], providing relatively favorable habitats for *A. leii*. Although prior studies have typically reported the negative effects of anthropogenic disturbances on firefly populations [45], the findings of the present study show a different pattern. This may be explained by the nonlinear effects of roads on insect communities [46]. Areas near roads may provide specific vegetation structures or temporary resources to support habitat use, resulting in variations in community composition and abundance. Alternatively, this association may also stem from spatial confounding between roads and stream networks. In the mountainous terrain of western Zhejiang, roads generally follow valley floors and watercourses, that means areas close to roads are usually adjacent to the aquatic habitats required by *A. leii* larvae. Therefore, distance to roads cannot be directly equated with the intensity of anthropogenic disturbance, and its effects may differ across insect taxa and landscapes.

### 4.2. Changes in Potentially Suitable Habitats

Under current conditions, the potentially suitable habitats of *A. leii* in the Zhejiang Province showed a pronounced spatial pattern characterized by aggregation in the west and sparsity in the east. Moderately and highly suitable habitats are primarily distributed continuously in the western region. Meanwhile, the eastern coastal plains and northern areas are dominated by low-suitability and unsuitable habitats. This pattern can be attributed to the hilly and mountainous landscapes in western Zhejiang, which help to maintain stream flow during dry periods and support larval development [47]. Under the future climate scenarios, the overall extent of potentially suitable habitats is projected to expand, whereas the proportion of highly suitable areas remains relatively low. This suggests limited adaptability of *A. leii* within its core habitat. Generally, species with narrow ecological niches are more constrained by environmental change [48], which may limit the capacity of *A. leii* to naturally colonize newly suitable habitats. Therefore, conservation measures such as habitat restoration or assisted dispersal may be required to promote population persistence and expansion.

Under the SSP126 scenario, potentially suitable habitats of *A. leii* primarily expand toward the eastern coastal regions of Zhejiang, where suitability is currently low. This pattern may be linked to the relatively low temperatures and unstable hydrological conditions under the present climate. Meanwhile, future warming may provide sufficient thermal conditions for larval development. In contrast, under SSP245, alterations in potentially suitable habitats are primarily reflected in localized expansion within western core habitats. These areas are characterized by complex topography and high vegetation cover, which can generate diverse microclimatic conditions and potentially buffer climate change effects [49]. Under SSP585, the spatial dynamics of potentially suitable habitats became more complex. High-suitability areas initially expand in Quzhou, Hangzhou, and Jinhua during the 2050s and then subsequently extend toward eastern coastal regions such as Ningbo and Zhoushan in the 2070s. This pattern may be associated with the intensified impacts of climate warming on mountain stream ecosystems under high-emission scenarios [50]. Considerable alterations in the water temperature and hydrological regimes may change larval survival conditions [51]. Therefore, long-term monitoring should be conducted in moderately and highly suitable habitats. Regions identified as suitable but lacking confirmed field records of *A. leii* should be prioritized for systematic surveys to verify their occupancy.

### 4.3. Spatial Associations Between Potentially Suitable Habitats and Landscape Patterns

The findings of the global bivariate spatial autocorrelation analysis indicate that potentially suitable habitats of *A. leii* have substantial spatial associations with multiple landscape metrics. With a few exceptions, potentially suitable habitats show positive correlations with NP, LSI, SHDI, and SHEI, whereas their relationship with LPI is variable. This suggests that *A. leii* tends to occur in heterogeneous landscapes characterized by high patch richness and complex edge structures, rather than in large, homogeneous patches dominated by a single landscape type. Such landscape configurations can form structurally complex ecotones that may provide diverse habitats and resources necessary across different life stages of *A. leii*. These four metrics capture distinct facets of habitat suitability for *A. leii* despite their strong covariation. The positive correlation with NP reflects that more dispersed patches can support multiple *A. leii* populations across the landscape. Meanwhile, the association with LSI highlights the value of complex aquatic-terrestrial edges for the species’ biphasic life cycle. The correlations with SHDI and SHEI further indicate that suitable habitats depend on both diverse land-use types and even patch distribution, rather than a single fragmentation gradient. The positive spatial associations between potentially suitable habitats and NP, LSI, and SHDI become progressively stronger with increasing greenhouse gas emissions and advancing projection periods. Therefore, under climate change, landscape complexity and heterogeneity play an increasingly crucial role in shaping the distribution of *A. leii*, and suitable habitats are more likely to be restricted to areas where heterogeneous landscape structures can buffer climatic stress [52].

Building on the spatial associations from global Moran’s I, we performed local analysis to detect heterogeneity not captured by global statistics. LISA cluster analysis showed that NP, LSI, SHDI, and SHEI, which are positively correlated with potentially suitable habitats, are spatially dominated by high–high clusters in the southwestern mountainous regions of Zhejiang. These areas, especially Lishui and Quzhou, are characterized by heterogeneous terrain and dense river networks, forming complex landscape mosaics that provide diverse microclimates and ecological niches, while improving ecosystem stability and resilience [53]. This supports *A. leii* persistence and reproduction. In contrast, the negative relationship between potentially suitable habitats and LPI shows two distinct spatial patterns. Low–high clusters predominate in the eastern coastal urbanized regions, indicating that *A. leii* tends to avoid large, homogeneous artificial landscapes such as continuous built-up areas or intensive agricultural land. This pattern is consistent with habitat preferences observed in several insect taxa [54,55]. In western Zhejiang, high–low clusters are more common, suggesting that suitable habitats are linked to dispersed small aquatic patches embedded within complex natural matrices. This configuration may reflect habitat fragmentation, with potential risks of population isolation even within core areas. Under future climate scenarios, these spatial association patterns generally remain stable, although shifts occurred under SSP585. Newly expanded suitable habitats tend to appear as isolated high–low or low–high clusters in southwestern Zhejiang. This suggests that climate change may increasingly force *A. leii* to occupy habitats within already fragmented landscapes. Based on these spatial characteristics, conservation strategies for *A. leii* should prioritize maintaining heterogeneous landscape structures with diverse patch types and complex habitat mosaics, and enhancing ecological connectivity among key aquatic and terrestrial habitats, particularly within current core habitats and newly suitable areas. Such measures can improve habitat resilience under climate change and support the persistence of *A. leii* populations.

While this study provides insights into climate change responses and habitat conservation of *A. leii*, some limitations remain. First, interspecific interactions were not incorporated into the distribution model, which may bias habitat suitability predictions. Second, the modelling framework relied on a single algorithm, which may not fully capture uncertainties associated with different modelling approaches. In addition, the model primarily reflects the ecological requirements of the adult stage of *A. leii*, while the aquatic larval stage was not fully considered. Third, spatial autocorrelation analysis primarily captures patterns of spatial association, whereas the underlying causal mechanisms linking landscape patterns to species distributions require further investigation. Nevertheless, our findings can form a foundation for future research in broader geographical and institutional contexts.

## 5. Conclusions

This study highlights the critical role of landscape patterns in shaping the response of *A. leii* to climate change. Soil properties and temperature are identified as the key environmental variables that affect habitat suitability. Suitable habitats currently occur mainly in western Zhejiang and may further expand under future climate scenarios. However, highly suitable areas remain limited and are becoming increasingly fragmented. Potentially suitable habitats exhibit strong spatial associations with landscape patterns, indicating a preference for heterogeneous landscapes with diverse and complex patch structures. Therefore, conservation strategies for *A. leii* should prioritize maintaining heterogeneous landscape structures with diverse habitat patches and complex landscape mosaics, while strengthening ecological linkages between aquatic and adjacent terrestrial habitats and prioritizing conservation in current core habitats and newly suitable areas to support long-term persistence. Incorporating biotic interactions and causal mechanistic analyses in future studies could further improve predictive accuracy and strengthen conservation guidance for *A. leii*.

## Figures and Tables

**Table 1:** Occurrence records of *Aquatica leii* in Zhejiang Province, China.

No.	East Longitude	North Latitude	No.	East Longitude	North Latitude
1	120.98	31.01	16	119.06	29.06
2	120.89	30.85	17	119.27	28.98
3	119.82	28.37	18	119.23	28.87
4	120.06	28.73	19	119.19	28.82
5	119.73	28.51	20	118.46	29.31
6	119.82	30.61	21	118.13	29.20
7	120.09	30.23	22	118.32	29.15
8	120.09	30.19	23	118.37	29.05
9	120.12	30.25	24	120.76	27.96
10	120.11	30.20	25	120.67	27.99
11	119.84	30.48	26	121.80	29.81
12	119.21	29.76	27	121.03	29.50
13	118.24	29.41	28	121.08	30.60
14	119.11	28.77	29	119.84	30.60
15	119.23	29.18	30	120.07	29.30

**Table 2:** Contribution and permutation importance of environmental variables (%).

Variable c|Ode	Environmental Variables Description	Contribution Rate	Permutation Importance
soil3	Soil clay content	19.2	15
bio1	Annual mean temperature	16.6	9.8
soil6	Soil pH	10.5	2.4
bio21	Slope	8.6	6.5
dr	Distance to roads	7.4	11.1
nl	Night light data	5.5	4.9
soil1	Soil sand content	5.5	13.3
bio2	Diurnal temperature range	4.4	0.2
NDVI	Normalized difference vegetation index	3.6	6.8
bio14	Precipitation of the driest month	2.5	3.1
bio22	Aspect	2.3	1.6
bio15	Precipitation seasonality	2.2	3.7
bio19	Precipitation of the coldest quarter	2.2	0.2
bio8	Mean temperature of the wettest quarter	2.2	2.5
bio17	Precipitation of the driest quarter	2.1	4.9
dw	Distance to water sources	1.6	0.6
soil4	Soil reference bulk density	1.2	10.2
soil2	Soil silt content	1.1	0.2
bio3	Isothermality	0.9	1
bio4	Coefficient of seasonal variation in temperature	0.2	2.1

**Table 3:** Selected landscape metrics.

Category	Metric	Abbreviation	Description
Area and edge	Number of patches	NP	Total count of discrete land-use patches within the landscape, where each patch represents a homogeneous habitat unit of the same land-use type.
	Largest patch index	LPI	Proportion of total area occupied by the largest patch
Shape	Landscape shape index	LSI	Normalized measure of edge complexity relative to landscape size
Diversity	Shannon’s diversity index	SHDI	Reflects compositional diversity based on patch type richness and proportion
	Shannon’s evenness index	SHEI	Indicates how evenly the area is distributed among patch types

**Table 4:** Pearson correlations of the selected landscape metrics.

	NP	PD	LPI	LSI	SHDI	SHEI
NP	1	0.769	−0.833	0.864	0.872	0.741
PD	0.769	1	−0.672	0.691	0.688	0.576
LPI	−0.833	−0.672	1	−0.898	−0.967	−0.948
LSI	0.864	0.691	−0.898	1	0.9	0.845
SHDI	0.872	0.688	−0.967	0.9	1	0.9
SHEI	0.741	0.576	−0.948	0.845	0.9	1

**Table 5:** Suitable habitat area of *Aquatica leii* under different periods and climate scenarios (km^2^).

Suitable Habitats	Comparison Indicator	Current	SSP126		SSP245		SSP585	
			2050s	2070s	2050s	2070s	2050s	2070s
Highly suitable habitat	Area Area change	6500 −	7800 1300	15,700 9200	20,400 13,900	15,100 8600	14,100 7600	23,900 17,400
Moderately suitable habitat	Area Area change	17,200 −	19,100 1900	28,100 10,900	29,400 12,200	26,200 9000	28,900 11,700	27,900 10,700
low suitable habitat	Area Area change	34,900 −	30,800 −4100	29,900 −5000	25,400 −9500	30,000 −4900	29,500 −5400	23,100 −11,800
Total suitable habitat	Area Area change	58,600 −	57,700 −900	73,700 15,100	75,200 16,600	71,300 12,700	72,500 13,900	74,900 16,300

Area change values are calculated relative to the current baseline.

**Table 6:** Local Indicators of Spatial Association (LISA) cluster of potentially suitable habitats and landscape metrics.

Climate Scenarios and Periods			SSP126		SSP245		SSP585	
		Current	2050s	2070s	2050s	2070s	2050s	2070s
NP	H-H	447	466	617	605	554	628	630
	L-L	590	397	547	549	503	610	466
	L-H	410	415	262	284	378	237	162
	H-L	344	570	407	433	474	326	346
	Not significant	3028	2971	2986	2948	2910	3018	3215
LSI	H-H	491	514	684	680	633	705	718
	L-L	529	352	506	529	483	571	426
	L-H	432	483	310	316	398	316	207
	H-L	248	491	300	350	396	270	308
	Not significant	3119	2979	3019	2944	2909	2957	3160
LPI	H-H	384	692	539	568	632	436	453
	L-L	419	385	269	357	366	262	213
	L-H	702	435	580	543	496	618	458
	H-L	365	456	531	475	463	547	464
	Not significant	2949	2851	2900	2876	2862	2956	3231
SHDI	H-H	564	582	749	725	664	757	723
	L-L	718	448	621	608	559	661	507
	L-H	489	526	333	388	463	333	212
	H-L	348	638	453	501	537	363	384
	Not significant	2700	2625	2663	2597	2596	2705	2993
SHEI	H-H	546	587	788	740	703	760	706
	L-L	650	406	574	551	511	609	466
	L-H	580	578	379	426	515	413	312
	H-L	319	573	393	444	484	333	378
	Not significant	2724	2675	2685	2658	2606	2704	2957

Values represent the number of grid cells classified into each LISA cluster type. NP, LSI, LPI, SHDI, and SHEI represent the number of patches, landscape shape index, largest patch index, Shannon’s diversity index, and Shannon’s evenness index, respectively. H–H, L–L, L–H, and H–L represent high–high, low–low, low–high, and high–low clusters, respectively.

## Data Availability

The original contributions presented in this study are included in the article. Further inquiries can be directed to the corresponding author.
